# Silicon Transporters and Effects of Silicon Amendments in Strawberry under High Tunnel and Field Conditions

**DOI:** 10.3389/fpls.2017.00949

**Published:** 2017-06-08

**Authors:** Samuel Ouellette, Marie-Hélène Goyette, Caroline Labbé, Joan Laur, Linda Gaudreau, André Gosselin, Martine Dorais, Rupesh K. Deshmukh, Richard R. Bélanger

**Affiliations:** Département de Phytologie–Faculté des Sciences de L'Agriculture et de L'Alimentation, Université LavalQuébec City, QC, Canada

**Keywords:** silicon, powdery mildew, day-neutral strawberry, *Podosphaera aphanis*, silicon absorption, Lsi1, Lsi2, silicon fertilization

## Abstract

Together with longer production periods, the commercial transition to day-neutral strawberry (*Fragaria* × *ananassa*) varieties has favored the development of diseases such as powdery mildew (*Podosphaera aphanis*) that thrives in late summer-early fall. In an attempt to find alternative solutions to fungicides currently employed to curb the disease, we wanted to investigate the potential of silicon (Si) amendments that have been associated with prophylactic properties against powdery mildews. To this end, our first objective was to determine if strawberry was a Si-competent species following the recent characterization of the properties of Si transporters that plants must carry to uptake silicic acid. Based on genomic data, we were able to conclude that strawberry contained both functional influx (Lsi1) and efflux (Lsi2) transporters for Si uptake. Subsequently commercial experiments under high tunnel and field conditions were conducted with different Si fertilization regimes: constant soluble Si feeding in high tunnel, and bi-weekly soluble Si feeding or three concentrations of calcium silicate fertilization in the field. Results from high tunnel experiments showed that strawberry could accumulate as much as 3% Si on a dry-weight basis, the highest concentration ever reported for this species. All six tested cultivars contained roughly the same concentration, thereby confirming the limited genetic variability, also observed in other species, associated with the trait. Silicon fertilization under high tunnel led to a significant reduction of powdery mildew severity in both years and on all cultivars, and a significant increase in yield of marketable fruits reaching as much as 300% with cv. Monterey. By contrast, Si fertilization under field conditions in soils deficient in plant available Si, either in soluble or solid form, did not result in significant accumulation of Si in plants, regardless of the cultivars, year or concentrations. Our results have thus provided both genotypic and phenotypic proof that strawberry can greatly benefit from Si fertilization, but have also highlighted the importance of validating the fertilization regime to ensure that Si is properly absorbed and/or available to the plant.

## Introduction

Strawberry has been exploited for its flavorful red fruits around the globe for centuries (Darrow, 1966). It is a perennial, dicotyledonous plant from the *Rosaceae* family (Maas, 1998). The commercial strawberry plant (*Fragaria* × *ananassa* Duchesne), is a hybrid species that came from the cross between *Fragaria chiloensis* and *Fragaria virginiana* (Hancock et al., 1999). There are three types of strawberry being cultivated today, and they are classified by their response to photoperiod. In June-bearing varieties, flowering is initiated by short days in the fall and a crop is produced the following year during spring-summer. They have been the standard strawberry type for many years in Canada until more productive varieties were developed. For instance, the everbearing type produces two crops, one in the spring and the other in the fall. More recently, the advent of day-neutral type, which corresponds to photoperiod insensitive plants, allowed fruit production through the fall as long as the temperatures are between 4° and 29°C (Dodgson, 2007). Since the 1990s, most strawberry growers use day-neutral varieties to produce fruits on longer periods and even on a year-round basis in Florida and California (Hancock and Simpson, 1995; Darnell et al., 2003).

Together with the longer production periods, the commercial transition to day-neutral types has favored the development of new diseases on strawberry. For instance, powdery mildew, a disease influenced by fluctuating humidity conditions in the late summer and fall months, has become a major problem (Dodgson, 2007). Powdery mildew on strawberry plants is caused by the fungus *Podosphaera aphanis*. It reduces the number of marketable fruits by decreasing fruit set, inducing cracks, and decreasing flavor and storage time (Pertot et al., 2008). It can reduce marketable yield from 20 to 70% (Dodgson, 2007). The optimal conditions for *P. aphanis* to thrive are long periods of temperatures between 18° and 22.5°C and an alternation between low and high relative humidity common in late summer- early fall (Amsalem et al., 2006). Since most day-neutral cultivars are susceptible to powdery mildew, additional treatments of fungicides are required to prevent and reduce the disease, leading to higher costs of production and potentially more pesticide residues on fruits (Xu et al., 2008). Also, several fungicides used against powdery mildew are part of quinone outside inhibitors (QoI) and sterol demethylation inhibitors (DMI). Both are known for their high resistance risk (Fernández-Ortuño et al., 2006; Sombardier et al., 2010). To circumvent this problem, other control methods are being sought to manage powdery mildew.

Over the last few years, several reports have highlighted the positive effects of silicon (Si) fertilization in agriculture (Liang et al., 2015a). Incidentally, the International Plant Nutrition Institute (IPNI) has recently added Si to its list of beneficial nutrients. For instance, several studies have shown that Si has prophylactic properties against a number of biotic and abiotic stresses on multiple crop species (Bélanger et al., 1995; Fauteux et al., 2005). Interestingly, Si is reported to be particularly efficient against diseases caused by biotrophic fungi such as powdery mildews (Vivancos et al., 2015). In the case of strawberry, very few studies have looked into the potential of Si either under experimental or commercial conditions. Wang and Galletta (1998) reported that foliar applications of Si significantly reduced strawberry powdery mildew (*P. aphanis*) and increased biomass but those results were contradicted by Palmer et al. (2006) who did not find any significant reduction of powdery mildew using foliar applications. On the other hand, Kanto et al. (2004, 2006) found that Si amendments in nutrient solutions and soils reduced the incidence of powdery mildew on strawberry.

Currently, Si fertilization remains marginal in strawberry in particular and in agriculture in general because there are still controversial and lingering questions regarding how plants can benefit from Si amendments, and methods of fertilization that optimize its effects. For instance, it is well recognized that silicic acid is the only soluble form a plant can absorb, but it is still unclear what plant species possess the adequate transport system, i.e., an influx and efflux transporter, to take up Si from the soil. No studies have ever looked into the presence of such transporters in strawberry so it remains unknown whether strawberry plants are good accumulators of Si. Accordingly, guidelines for Si fertilization in strawberry crops are lacking.

Considering the increasing presence and pressure of powdery mildew on day-neutral strawberry varieties, and the potential to exploit Si as an alternative to synthetic fungicides to control the disease, we were interested to determine if strawberry was a potential accumulator of Si and the fertilizing conditions that would optimize its absorption. In this context, our objectives were: (1) to analyze genomic data from strawberry for the presence of *bona fide* Si influx transporter, belonging to the family of NIP-III aquaporins as per Deshmukh et al. (2015), and Si efflux transporter; (2) to evaluate soluble and/or solid forms of Si fertilizers under both high tunnel and field commercial productions on several day-neutral varieties; and (3) to determine and compare Si accumulation, powdery mildew incidence and fruit yield for all varieties under the different fertilization treatments. Our results show for the first time that strawberry has the genetic and phenotypic predisposition to absorb Si but that Si fertilization regimes will influence the amount absorbed by the plants and thus the benefits they derive from Si.

## Materials and methods

### Presence of Si transporters in strawberry

Plant species capable to accumulate Si must contain influx transporters (Lsi1) of the type NIP-III aquaporins and efflux transporters (Lsi2), known as putative ion transporters (Ma and Yamaji, 2015; Deshmukh and Bélanger, 2016). Genomic data were analyzed for the presence of such transporters as previously described in Shivaraj et al. (2017). Protein sequences from the FAN_r1.1 and FANhybrid_r1.2 versions of *Fragaria* × *ananassa* reference genome were retrieved from the GDR (Genome Database for Rosaceae, https://www.rosaceae.org/). Blast search performed using the query sequences of known NIP-IIIs and Lsi2 homologs from soybean, poplar, wheat and rice against both versions only identified partial NIP-III and Lsi2 homologs. Consequently, assembly of available RNA-seq data for *Fragaria* × *ananassa* tissues from SRA database (https://www.ncbi.nlm.nih.gov/sra) along with FAN_r1.1 were used to obtain full sequences.

### Construction of phylogenetic tree, protein 3D structure and transmembrane domain profiling

Phylogenetic trees for NIP-IIIs (Lsi1s) and Lsi2s were constructed using MEGA (version 6) software tool. Protein sequences were aligned using ClustalW and subjected to construct phylogenetic tree using Maximum likelihood method with 1000 bootstrap iterations. The protein 3D structures were constructed using the SWISS-MODEL server with an automated protein homology-modeling option (https://swissmodel.expasy.org/). The transmembrane domain profiling was performed using TMHMM tool (www.cbs.dtu.dk/services/TMHMM/). Functional annotation of Lsi2 was performed with Conserved Domain Database (CDD, www.ncbi.nlm.nih.gov/Structure/cdd/cdd.shtml).

### Location of commercial experiments

The experiments under high tunnel were conducted at the farm of Les Fraises de L'Île d'Orléans inc. (St. Laurent, Ile d'Orléans, Québec, Canada) from May to October 2014, and 2015. A high tunnel of 30 × 8 m with a simple polyethylene plastic membrane was used and the sides of the tunnel were open to allow for ventilation.

The field experiments were conducted at the Ferme François Gosselin (St. Laurent, Ile d'Orléans, Québec, Canada) from May to October 2015, and 2016. For both seasons, plants were placed on raised beds covered with a plastic mulch in mid-May. Space between rows was 1.3 m while space between plants was 28 cm.

### Plant material and soils

#### High tunnel experiments (2014 and 2015)

A set of 6 day-neutral (DN) cultivars namely Charlotte, Seascape, Monterey, Albion, Amandine and Verity were used in this study. The first three were used in 2014 and all six in 2015. Charlotte is a cross from Mara des bois and CAL 19 and was created in 1995 in France. It has good hardiness and resistance to powdery mildew. Seascape is a favorite among commercial growers but is considered susceptible to powdery mildew. Albion is a cultivar known for its large, glossy, and tasty fruits, but there are no reports on its susceptibility to powdery mildew. The offspring of Albion, Monterey, is a relatively new cultivar with large fruits, but with fairly high susceptibility to powdery mildew. Amandine is a new cultivar produced in Spain with a high-yielding potential and a good resistance to powdery mildew. Verity is also a new cultivar bred in the United Kingdom. Its interaction with powdery mildew is unknown but it has a high yield potential with glossy fruits. All plants were transferred to production in mid-May for both years and were grown in a soil-less substrate, Mélange Bio (Fafard & Frères, Qc).

#### Field experiments (2015 and 2016)

In 2015, the experiments were conducted at l'Ile d'Orléans at La Ferme François Gosselin on an Orleans type soil being in the brunisolic order and the great group dystric brunisol (Marcoux, 1980). To determine Si levels in soil, the calcium chloride extraction method was used (Liang et al., 2015b). The soil pH was 6.4 and the plant available Si ranged between 19 and 21 mg/kg, a concentration considered very low since concentrations below 100 mg/kg are considered deficient (Liang et al., 2015b). The cultivars Seascape (DN), San Andreas (DN) and Jewel (June bearing) were tested.

In 2016, the experiments were conducted in fields from the same grower where the soils were in the podzolic order and in the great group of humo-ferric podzols. Their pH was 6.7 and their plant available Si content was measured between 15 and 16 mg/kg. The cultivar San Andreas was replaced by Albion (DN). For each of the 2 years, bare-root plants obtained from Lassen Canyon Nursery Inc. (Redding, CA) were transferred into the field in early May.

### Experimental design

#### High tunnel experiments (2014 and 2015)

The experimental design was a randomized block design. For the first year (2014), we tested three cultivars (Seascape, Charlotte, and Monterey) in combination with the two fertilization regimes (control and Si+) that were distributed in four blocks as repetitions of the six treatments. In the second year (2015), we had the same two fertilization regimes (control and Si+) in combination with six cultivars (Seascape, Charlotte, Monterey, Albion, Amandine, and Verity) in four blocks. In both experiments, each gutter supporting the pots was treated as a block. For each Si treatment and cultivar in each block, five pots containing three strawberry plants were used.

#### Field experiments (2015 and 2016)

In 2015, two fertilization regimes (control and Si+) were used in combination with three cultivars in a split-plot design where three blocks enclosed the Si treatment as the main plot and cultivars (3) as subplots. Plant number for each combination was 20 and statistical analysis was conducted on three (N) independent values for yield calculation. In 2016, four Si treatments were used with three cultivars. The experimental design was randomized complete blocks (6) with eight plants for each cultivar, each treatment in each block.

### Silicon treatments and fertilization

#### High-tunnel experiments (2014 and 2015)

Two 1,000-l containers were used for holding the different solutions that fed the plants by a drip feed irrigation. The rate of the drip irrigation was 4 l per pot per hour. The water tension was continuously recorded with a tensiometer. The irrigation was manually activated when the water tension was below −3.0 kPa. For the Si+ treatment, liquid potassium silicate (Kasil®, PQ Corporation) was used in one of the 1,000-l container to obtain the maximum soluble concentration of 1.7 mM Si while the other had the control treatment (Si-) and an additional K (0-0-52) to compensate for the addition of K in the container with Kasil®, Since the Si amendment was directly added to the nutrient solution, Si was fed to the plants whenever irrigation was applied (typically 20 min a day). Over the experimental period, each plant received on average 800 ml of solution per week.

During the experiments, volume, pH, and EC of the drainage solutions were measured daily in order to correct the nutrient solutions in case of mismanagement. Air temperature, soil temperature and air relative humidity were recorded continuously with a HOBO™ data logger.

#### Field experiments (2015 and 2016)

In 2015, soluble Si in the form of Kasil® was used as Si source. Briefly, a 1,000-l tank was used to prepare a solution 1.7 mM Si for which the pH was adjusted to 7 with nitric acid. The control solution (Si-) was amended with (13-0-46) to counterbalance K and N brought by Kasil® and the nitric acid in Si+ solution. Based on the grower's estimate of fungicide costs, Si applications were made so that the cost of Si fertilization would not exceed that of fungicides. Accordingly, both the Si and control treatments were applied twice a week by drenching with a drip tape system (AquaTraxx) previously installed. Each fertigation, with a duration of 60 min, gave *ca*. 620 ml of solution per plant per week.

In 2016, wollastonite (58% SiO_2_, 23% CaO, 6% MgO) (Canadian Wollastonite, Kingston, ON), mesh size 400, was used as Si source based on reports (Liang et al., 2015a), preliminary experiments in the greenhouse and its high level of plant available Si. Four doses based on conversions from pot experiments were tested: 0 (control), 12, 24, and 36 g per plant corresponding roughly to 2,000, 4,000, and 6,000 kg per ha, respectively, with the former being a common concentration for field applications (Datnoff et al., 1997). At the planting stage, the wollastonite was incorporated directly into the planting holes and bare root strawberry plants were immediately planted. Fertilization was performed according to the grower's standard regime in all treatments.

### Silicon accumulation in strawberry leaves and fruits

To measure Si concentrations in plants, the oldest leaves of the plants were sampled, dried at 60°C for at least 2 days and pulverized with a bead mill homogenizer (Omni Bead Ruptor 24, Omni International). Silicon concentrations were measured with the X-ray fluorescence spectrometry method (Niton XL3t955 GOLDD+ XRF) adapted from Reidinger et al. (2012). Leaf sampling was made in mid-July and mid-September for high-tunnel experiments and in mid-September for field experiments. In parallel, fruits were sampled regularly over the sampling period and stored for measurements of Si concentrations.

### Powdery mildew severity

The incidence of powdery mildew was evaluated every week with a disease scale adapted from Horsfall and Barratt (1945). This scale consisted to evaluate a global infection level of the leaves for a plant where 0 meant no powdery mildew and 5 meant more than 75% of the entire plant presenting symptoms and/or signs.

The AUDPC (area under the disease progress curve) was used in order to quantify the disease severity:

$$AUDPC=\sum_{i=1}^{N_{i-1}}\frac{\left(y_{i}+y_{i+1}\right)}{2}\left(t_{i+1}-t_{i}\right)$$

where y = disease level

and t = time of record

### Yield and fruit quality

For both, tunnel and field experiments, yield and fruit quality were measured three times a week from mid-June until the end of September. The variables for measuring yield was the weight of marketable fruits per plant. Fruits were considered unmarketable if too small (less than 6 g) and/or misshapen and/or diseased.

### Data analysis

ANOVA was performed on data with the software JMP version 12.0.1 (SAS Institute Inc.). When significant (*p* < 0.05), additional statistical tests were performed. Tukey was used for multiple comparison when allowed. When indicated in the figure legends, orthogonal contrasts were also used and a *P* < 0.05 was considered statistically significant.

## Results

### Presence of influx and efflux silicon transporters in strawberry

**Figure 1:**
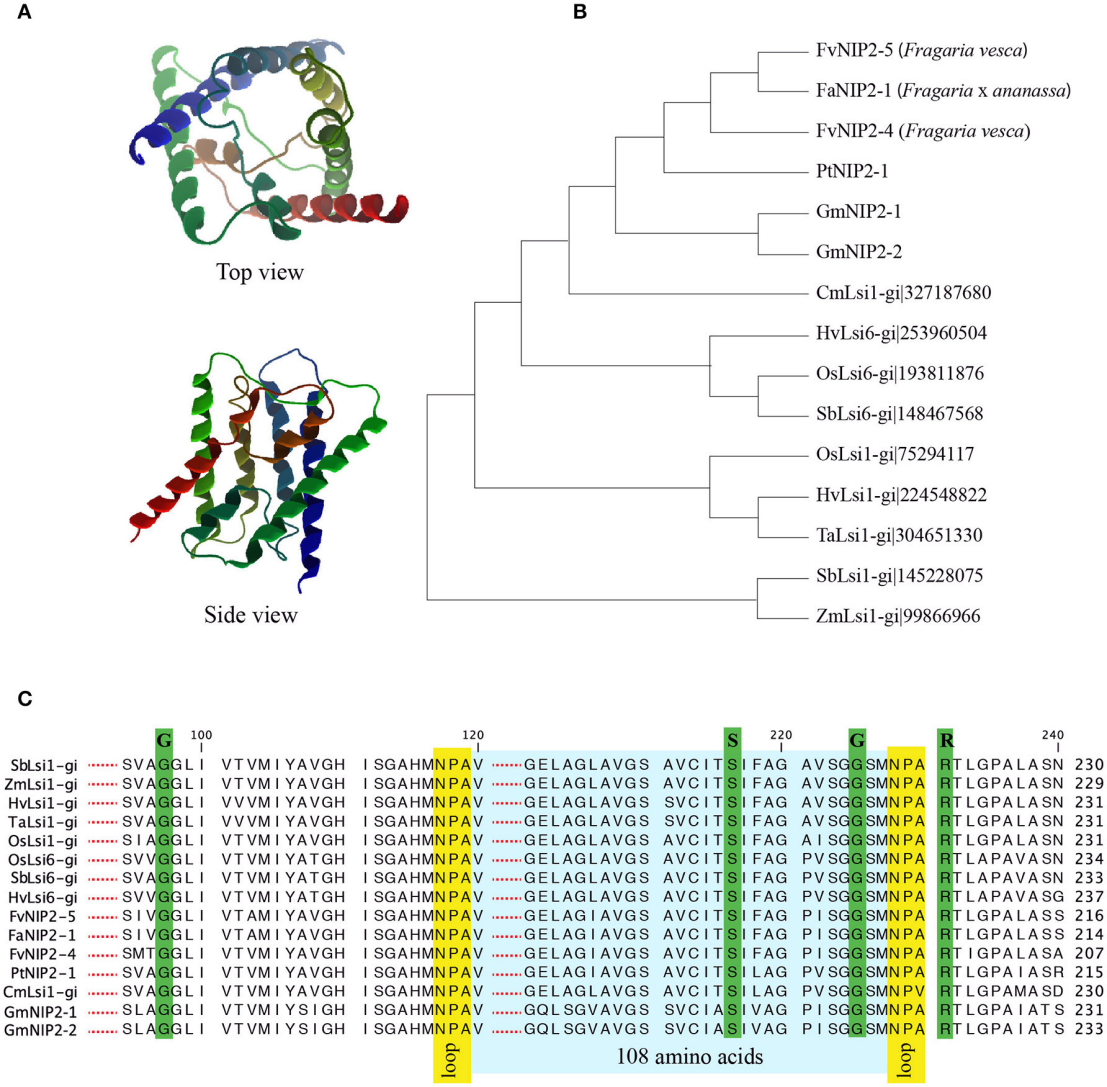
**(A)** Three-dimensional structure of putative silicon transporters in strawberry; **(B)** Cladogram of NIP2 from *Fragaria* spp. with confirmed silicon transporters from other plant species; **(C)** Alignment of NIP2s, from *Fragaria* spp., with sequences of confirmed Lsi1. The entire protein sequences are provided in Supplementary Data sheet 1. Fa, *Fragaria* × *ananassa*, Fv, *Fragaria vesca*, Pt, *Populus tremuloides*, Gm, *Glycine max*, Cm, *Cucumis melo*, Hv, *Hordeum vulgare*, Os, *Oryza sativa*, Sb, *Sorghum bicolor*, Ta, *Triticum aestivum*, Zm, *Zea mays*.

A single influx (Lsi1) Si transporter, FaNIP2-1 belonging to NIP-III, was identified in strawberry. The FaNIP2-1 showed the characteristic features reported to be required for the functionality of the protein with respect to Si permeability (Figure 1). The FaNIP2-1 has six transmembrane domains, two conserved NPA motifs, a G-S-G-R Ar/R selectivity filter and, importantly, the 108 AA spacing between NPA motifs (Figures 1A,C). Furthermore, homology-based 3D protein model of FaNIP2-1 showed a typical hourglass-like structure (Figure 1A). Detailed investigation of FaNIP2-1 protein structure revealed a pore formed at the center of the protein making a transmembrane channel capable to transport Si (Supplementary Figure 1). Phylogenetic analysis clustered FaNIP2-1 along with influx Si transporters from other dicot species including wild strawberry, cucumber, and soybean (Figure 1B). Protein sequence alignment of FaNIP2-1 with known Si transporters showed conserved Ar/R selectivity filters, NPA motifs and the spacing between NPA domains (Figure 1C).

**Figure 2:**
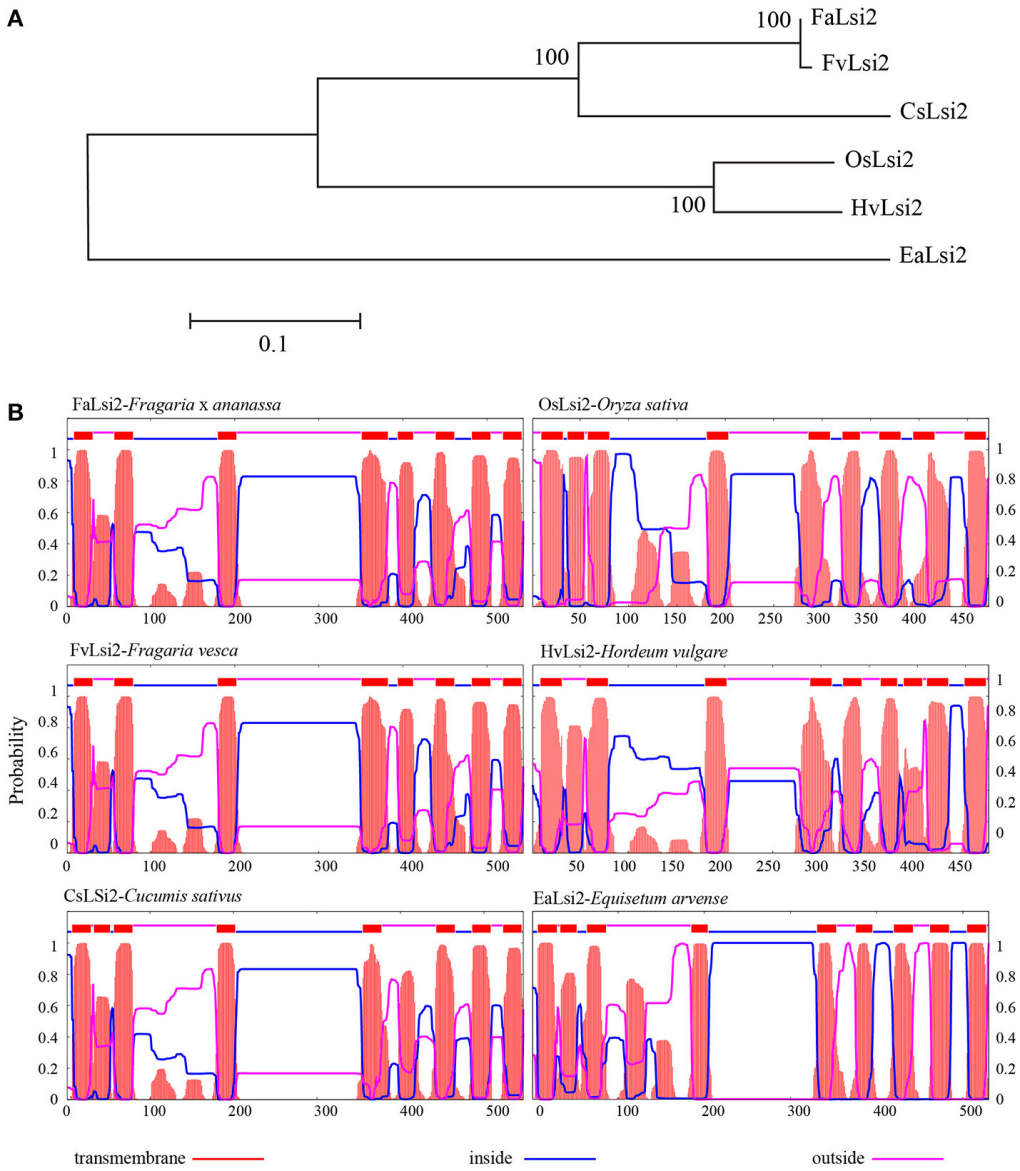
**(A)** Phylogenetic relationship between the efflux silicon transporter (FaLsi2) identified in *Fragaria* × *ananassa* (Fa) and other plant species and **(B)** distribution of the 11 transmembrane domains predicted in *Fragaria* × *ananassa* protein with those of *Fragaria vesca* (Fv) and the four functional Si efflux transporters previously identified in four plant species, predicted with TMHMM tool (http://www.cbs.dtu.dk/). The entire protein sequences are provided in Supplementary Data sheet 1. Cs, *Cucumis sativus*, Hv, *Hordeum vulgare*, Os, *Oryza sativa*, Ea, *Equisetum arvense*.

Similarly, a single efflux (Lsi2) Si-transporter was observed in strawberry. Functional classification of proteins via subfamily domain architectures performed using CDD search classified the protein as a Si transporter (Supplementary Table 1). It showed typical transmembrane domain profile as observed in known Lsi2s from different plant species (Figure 2).

### Silicon concentration in leaves and fruits

#### High-tunnel experiments

**Figure 3:**
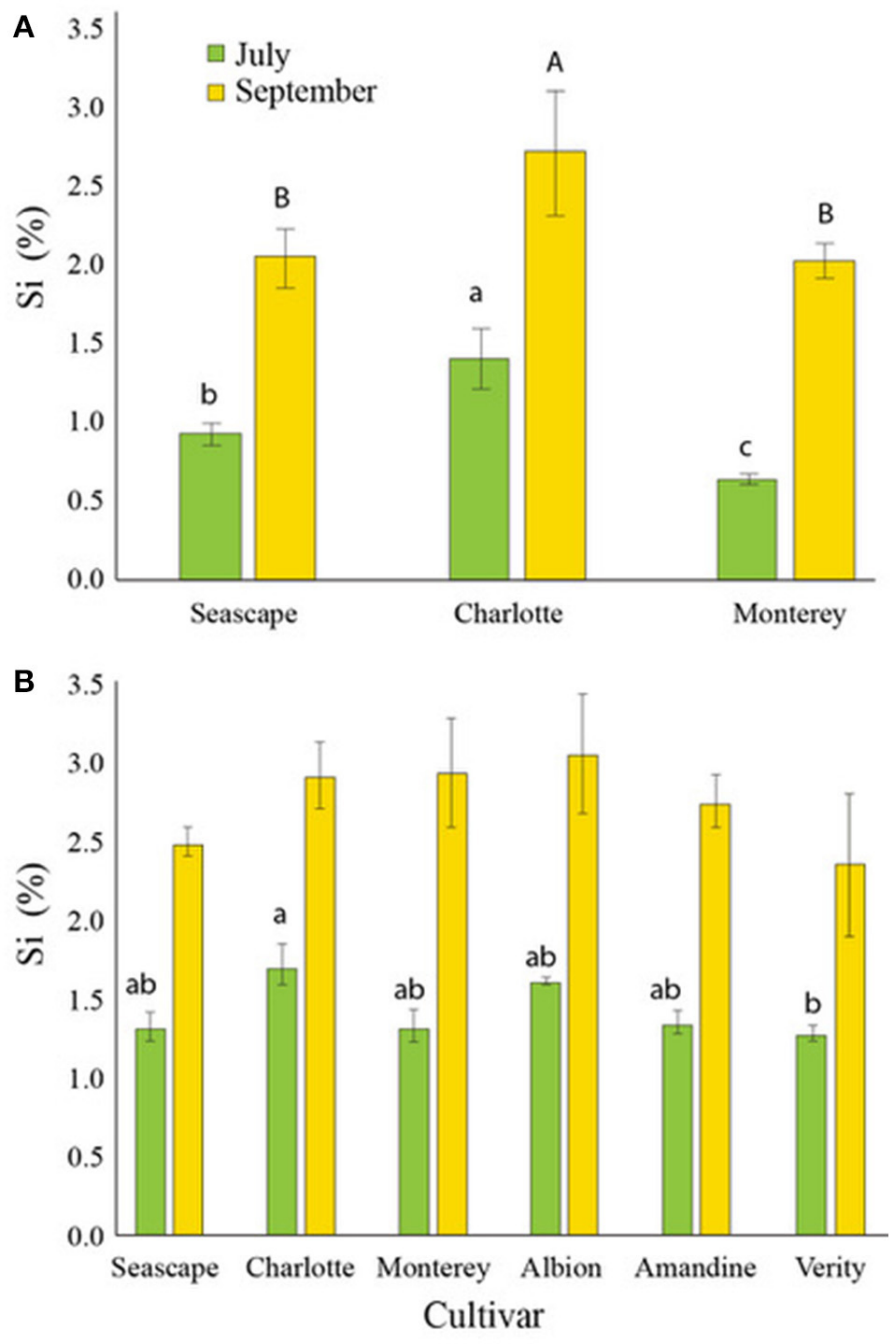
Percent average silicon (Si) content in leaves of strawberry plants treated daily with a 1.7 mM Si solution in a high-tunnel commercial setting for two different dates in summer 2014 **(A)** and in summer 2015 **(B)**. Each value represents the mean ± SE (*n* = 4). Multiple comparisons between cultivars were made each month independently. Means with different letters are significantly different (Tukey HSD).

In control nutrient solutions, strawberry plants accumulated 0.42% ± 0.07 Si d.w. in their leaves by mid-July, and 0.72% ± 0.13 by the end of September, regardless of the cultivar or the year (data not shown). By contrast, plants fertilized with Si accumulated close to or more than 1% Si d.w. by July and more than 2% d.w. by mid-September (Figures 3A,B). In 2014, Charlotte had significantly more Si than the other cultivars under study at both sampling times (Figure 3A).

In 2015, analysis of strawberry leaves of all six cultivars showed that they all accumulated between 1.0 and 1.5% Si d.w. with only a significant difference between Charlotte and Verity by mid-July (Figure 3B). Toward the end of the season, all cultivars had concentrations ranging between 2.5 and 3.0% d.w. No significant differences in Si concentrations were observed among the cultivars in September.

Strawberry fruits harvested throughout the season were measured for Si concentration. In all tested samples, Si concentrations never reached the level of detection (LOD) indicating that Si was never translocated to the fruit (data not shown).

#### Field experiments

**Figure 4:**
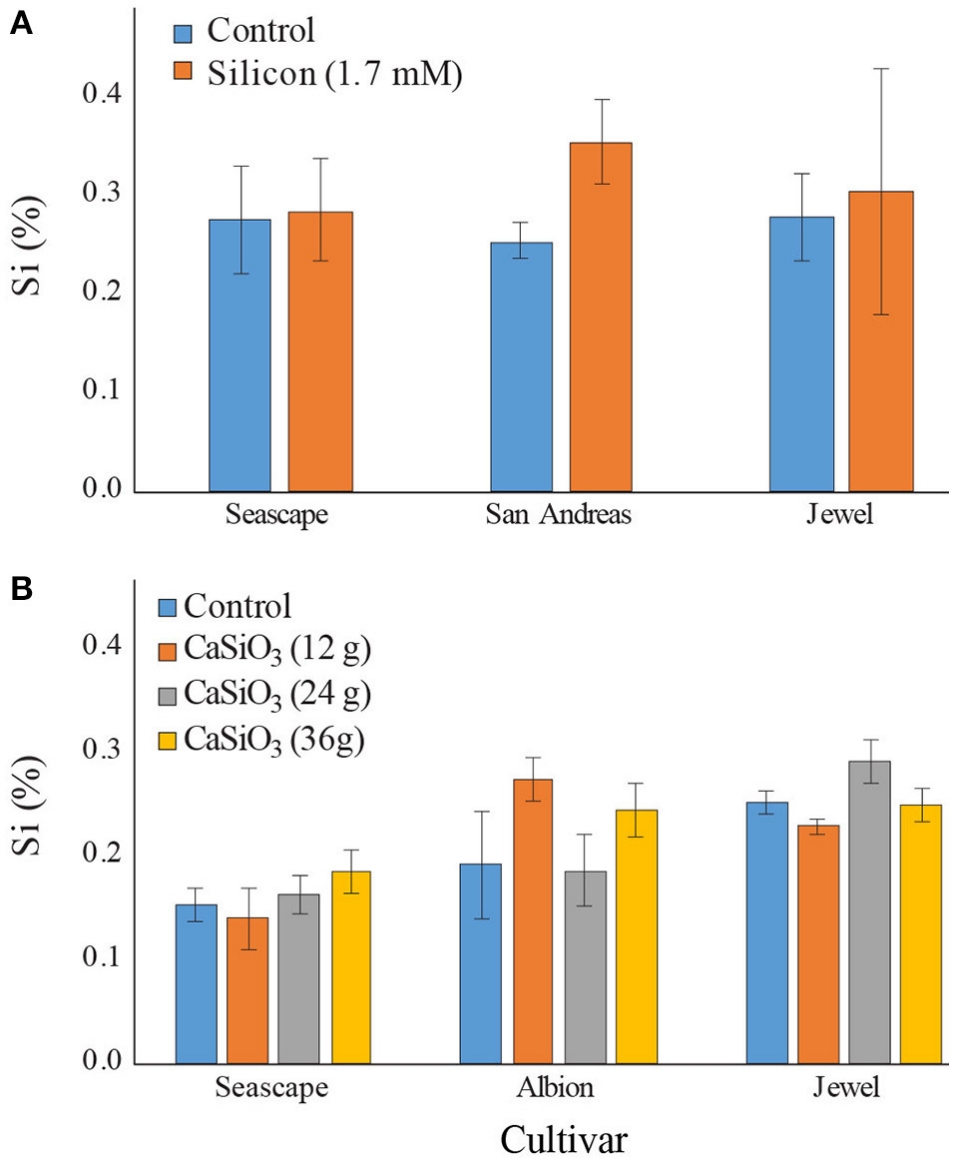
Percent average silicon (Si) content in leaves of strawberry plants grown in field **(A)** treated twice a week with a 1.7 mM Si solution in summer 2015 **(B)** or fertilized with different concentrations of calcium silicate (wollastonite) in summer 2016. Sampling was made in September for both experiments. Each value represents the mean ± SE (*n* = 3 in 2015; *n* = 6 in 2016).

In the course of field experiments, the Si concentrations were markedly different than the ones observed in high tunnel experiments. Control plants never exceeded a concentration of 0.3% in either 2015 or 2016 for all cultivars tested (Figures 4A,B). Surprisingly, a bi-weekly fertilization of Si with potassium silicate did not significantly increase Si concentration in plants by mid-September (Contrast _(*Si vs*. *Control*)_; *P* = 0.4846) (Figure 4A). Only plants from San Andreas appeared to accumulate more Si as a result of the Si treatment. Plant Si levels obtained in the field were nearly 8 times lower that the ones observed in high tunnel experiments.

In 2016, calcium silicate as a solid source of Si fertilization did not improve Si intake by the plants regardless of the concentrations used (Contrast _(calcium silicate doses)_; *P* = 0.6184) (Figure 4B). The concentrations *in planta* never exceeded 0.3%, in stark contrast once again with observations from the high tunnel experiments.

### Powdery mildew severity

#### High tunnel experiments

**Figure 5:**
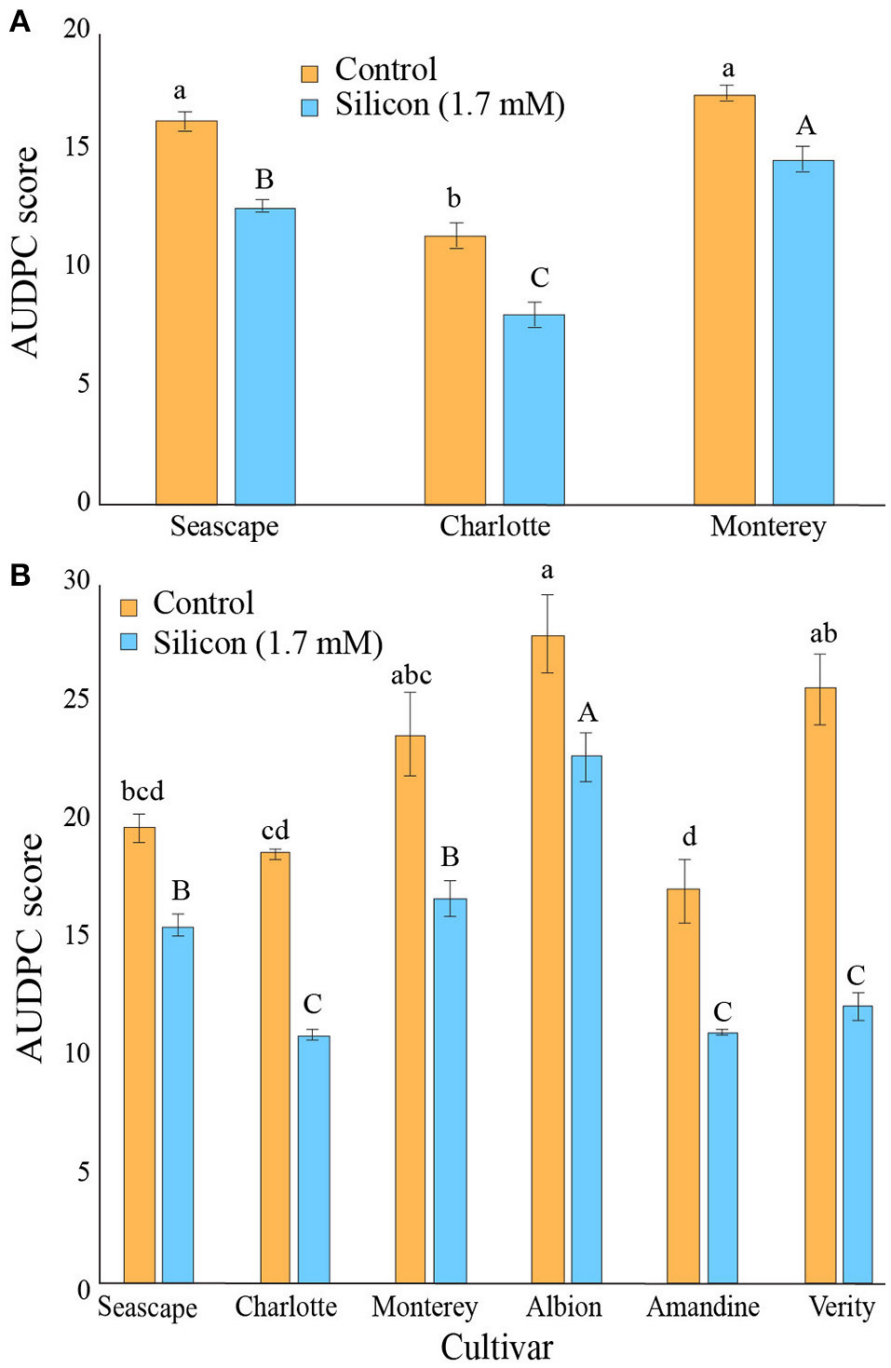
Area Under Disease Progress Curves for *Podosphaera aphanis* on strawberry leaves of plants treated or not with 1.7 mM Si in a high tunnel commercial setting during summer 2014 **(A)** and summer 2015 **(B)**. Each value represents the mean ± SE (*n* = 4). Multiple comparison test (Tukey HSD) has been done on each treatment independently. Means with different letters are significantly different.

In high tunnel experiments, the first signs of powdery mildew appeared in mid-July and expanded through September in both years. Figure 5A shows the average AUDPC calculated from the global infection level values observed on strawberry leaves during the growing season for the three cultivars treated or not with Si in 2014. The Si treatment consistently provided better disease control on all three cultivars (Contrast _(Si vs.Control)_; *P* < 0.0001) with a reduction of *ca*. three units of AUDPC. Plants from cv. Charlotte were naturally more resistant than those of cvs. Monterey and Seascape (Figure 5A).

In 2015, powdery mildew severity was slightly higher than in 2014. As a result, the prophylactic effects of the Si treatment were even more apparent, and significant reduction of powdery mildew severity was observed on all six treated cultivars (Contrast _(Si vs.Control)_; *P* < 0.0001). In particular, plants from cvs. Charlotte, and Verity responded to Si fertilization with reductions of AUDPC score ranging from 40 to 50% (Figure 5B).

#### Field experiments

In field experiments, powdery mildew severity was nearly absent in both 2015 and 2016. Therefore, no differences could be observed among the cultivars or between the Si treatments.

### Yield and fruit quality

#### High tunnel experiments

**Figure 6:**
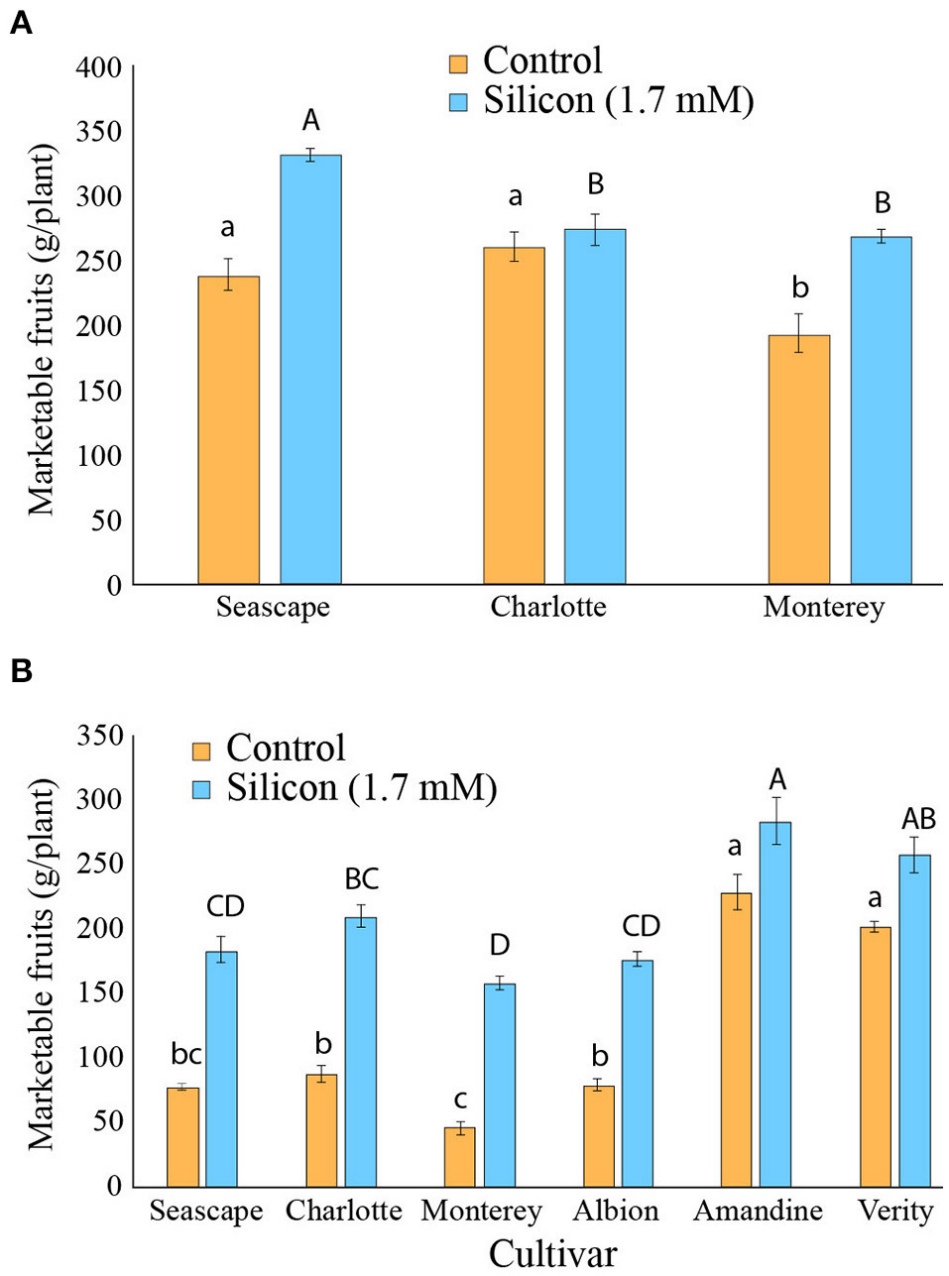
Weight of marketable fruits for strawberry plants treated or not with potassium silicate in a high tunnel commercial setting. **(A)** Summer 2014. **(B)** Summer 2015. Each value represents the mean ± SE (*n* = 4). Multiple comparison test (Tukey HSD) has been done on each treatment independently when the ANOVA was significant. Means with different letters are significantly different.

During the course of the high tunnel experiments, fruits were harvested and graded as marketable or not throughout the growing season. In 2014, a significantly higher yield of marketable fruits was obtained as a result of Si fertilization (Contrast _(Si vs.Control);_ *P* < 0.0001; Figure 6A).

In the 2015 experiments, where powdery mildew severity was higher, the beneficial effects of Si on marketable fruits per plant were strikingly apparent on all cultivars tested (Contrast _(Si vs.Control)_; *P* < 0.0001). While yields were generally lower in 2015, differences between control and Si+ plants were sometimes as high as 300%, as in cv. Monterey for example (Figure 6B). In general, the cultivars that showed the lowest yields for marketable fruits in control plants in 2015, e.g., Seascape, Charlotte, Monterey, and Albion, were the ones that benefited the most from Si fertilization (Figure 6B). Although the Si effect was more modest for Amandine and Verity plants, these two cultivars showed the overall highest yield under Si treatment (Figure 6B). No sign of phytotoxicity or albinism on fruits was ever observed during the experiments.

#### Field experiments

**Figure 7:**
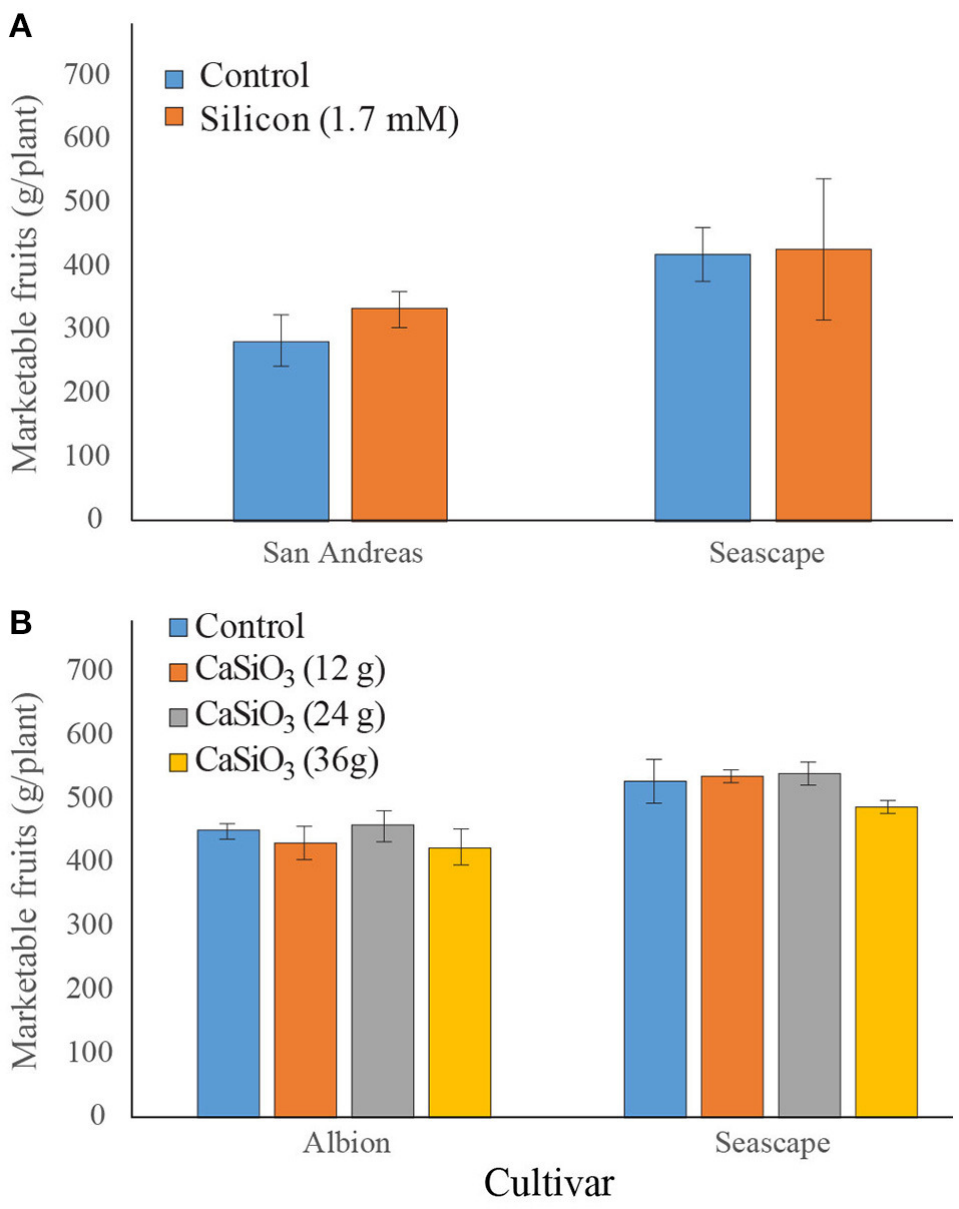
Number of marketable fruits on strawberry plants treated or not twice a week with a 1.7 mM Si solution **(A)** in the field for summer 2015 or treated or not with different concentrations of wollastonite **(B)** in summer 2016. Each value represents the mean ± SE (*n* = 3 in 2015; *n* = 6 in 2016).

In field experiments, no significant difference was observed in marketable fruits as a result of a bi-weekly fertilization with soluble Si (Figure 7A). On the other hand, plants from cv. Seascape appeared to be slightly more productive than those of cv. San Andreas (Contrast _(cvs)_; *P* = 0.0562). In 2016, when plants were subjected to a solid source of Si fertilization, no difference in yield was observed under Si treatment (Figure 7B) but plants from Seascape outperformed those of Albion (*P* < 0.0001). No yield data are available for Jewel because it is a short-day cultivar that only initiates its flower buds in the fall for production the following season.

## Discussion

This study provided the first genetic proof that strawberry possesses both influx (Lsi1) and efflux (Lsi2) Si transporters and, as a result, is a receptive plant for Si fertilization. Our results further showed that the fertilization regime will greatly influence the benefits strawberry derives from the treatment.

In spite of the growing interest for Si fertilization in agriculture, many plant species are unable to absorb Si to concentrations that will yield benefits. For the longest time, plants were categorized as low, intermediate and high Si absorbers based on empirical data (Jones and Handreck, 1967), with the latter two categories being seemingly the only ones where Si was associated with positive effects. The discovery of Si transporters in rice (Ma et al., 2006, 2007) offered the first scientific basis to determine with precision what plant species could absorb Si. Indeed, Deshmukh et al. (2015) recently showed that plants possessing NIP-III aquaporins with the proper configuration for Si were able to absorb Si to concentrations exceeding 1% and that all other species lacking this specific transporter were unable to actively take up Si from the soil. Previous works offered conflicting data about the predisposition of strawberry for Si absorption (Miyake and Takahashi, 1986; Kanto et al., 2006) and the latest classification by Hodson et al. (2005) suggested that strawberry was a poor absorber. We thus took advantage of available genomic data to ascertain if strawberry did possess Si influx and efflux transporters. Our results clearly showed that it contained an influx transporter with a GSGR pore and a 108 aa distance between the NPA loops as essential conditions for Si uptake. We further showed that it also contained a Lsi2 with high-sequence and transmembrane profile similarities to functional Lsi2s reported in rice, barley, cucumber and horsetail (Vivancos et al., 2016). On the basis of this evidence, we concluded that strawberry could be classified as a Si-competent species, which supported our initiatives to conduct field trials.

Large-scale Si fertilization of strawberry under two commercial settings, high tunnel and field, highlighted interesting yet unexpected results. In the high tunnel experiment, where Si was fed to the plant in soluble form on a regular basis, we have shown that strawberry can accumulate Si in concentrations as high as 3%, the highest level ever reported for this species. This result corroborates perfectly our genomic data and should eliminate all confusion with respect to the classification of strawberry as Si absorber. All six cultivars tested over the course of the two experimental seasons accumulated roughly the same concentration, which would indicate a limited genetic variability for the trait, at least within the germplasm under study. It is noteworthy to mention that, to date, very little within-species variability has been observed among plants in terms of Si accumulation. This suggests that Si permeability is tightly controlled by the transporters Lsi1 and Lsi2, with very little flexibility for intermediate potential, as shown by Deshmukh et al. (2015). Interestingly, our results confirmed that Si was not translocated to the fruit, thereby eliminating concerns about Si potentially affecting fruit firmness or quality.

In contrast to the high-tunnel experiments, we observed concentrations averaging only 0.3% Si in plants of field experiments treated with soluble Si twice a week. Except for a marginal increase of 0.1% with cv. San Andreas, the treatment did not increase Si concentrations in plants over a 4-month period. These results are surprising considering that field plants only received 22.5% less Si solution than high tunnel plants (620 vs. 800 ml). Assuming perfect absorption of over 500 mg Si delivered to the plant over the course of the experiment, this could have translated into over 2–3% Si, on the basis of dry plants averaging between 15 and 20 g. One possible reason can be leaching of the solution although we would have expected at least some increments considering the solution was delivered directly at the base of the plant. Another possibility is that silicic acid became unavailable to the plant because of an interaction with soil particles. It is well known that some soils will release more or less silicic acid depending on texture, pH, conductivity, although this phenomenon has been less described with soluble fertilizers (Liang et al., 1994; Tubana et al., 2016). Nevertheless, further investigations are warranted to determine if our liquid or solid fertilization treatments actually increased plant available Si in the treated soils, and how soil properties can influence this important parameter.

Following the poor results obtained in 2015, we decided to test Si fertilizers that are more affordable and easier to apply. Calcium silicates are by far reputed to be the best source for Si fertilization with wollastonite being a particularly good substrate of plant-available Si (Pereira et al., 2004). At the end of the summer, regardless of the cultivar tested, or the calcium silicate concentration added to the plants, our results once again showed low Si concentrations below 0.25% and no significant differences in line with the Si treatment. These results raise a number of questions while answering some of the inconsistencies reported in the literature with respect to Si fertilization. In terms of questions, it is indeed puzzling that no increase in plant Si concentrations was observed, especially since preliminary experiments in soilless media had suggested that wollastonite would be a good source of Si (unpublished results). From the start, both soils where experiments took place were very low in plant available Si, a result well in line with concentrations found in control plants. This could mean that they had low Si content initially, but more importantly, that they were not conducive for silicic acid solubilization. For instance, light soils, generally sandy soils such as ours, do not have a good content in Si availability. Liang et al. (1994) related that the correlation with Si availability is positive with clay content but negative with sand content. In addition, in the case of a neutral soil (pH > 6.5), the silt content, the sand content and the pH are three factors negatively correlated with Si availability (Liang et al., 1994). In the field experiments, pH was 6.4 (2015) and 6.7 (2016) whereas soil texture was silty-sandy-loam (2015) and sandy-loam (2016), conditions that correlate well with the low Si concentrations found. Our results further suggest that Si amendments in such soils are not conducive to release silicic acid. Incidentally, Kanto et al. (2006) did not find accumulation of Si in strawberry plants fertilized in the field, although soil properties were not reported. On the other hand, in high tunnel experiments and in preliminary greenhouse experiments, plants grew in sphagnum-peat-moss substrate, which is known to be very acidic (Payette and Rochefort, 2001). This would further support that acidic pH conditions are more amenable to Si fertilization as they promote a better release and solubilization of silicic acid.

Our data can serve to explain the inconsistencies and confusion often encountered in the literature with respect to Si benefits. As a first point, the disparities in Si concentrations we observed between high tunnel and field strawberries explain why the classification of strawberry, in terms of Si absorption, has been a source of conflict on the basis of phenotypes. As a second point, it brings into question the reliability of Si fertilizers and data reporting or not benefits. Unfortunately, most papers studying Si fertilization fail to report the amount absorbed by strawberry plants (Dehghanipoodeh et al., 2016; Yaghubi et al., 2016). The consequences of these oversights are that faulty conclusions can be drawn, leading to negative reports about Si benefits, attributable only to a deficient treatment, or positive results, attributable to other properties of the fertilizers.

In high tunnel experiments where Si fertilization led to high Si concentrations in plants, significant reductions in levels of powdery mildew severity were observed in all cultivars tested in both in 2014 and 2015. Interestingly, cv. Charlotte, reputed for its resistance to *P. aphanis*, did have the lowest powdery mildew severity among tested cultivars in both seasons, and in both the control and the Si+ treatments. These results confirm the multiple reports of the prophylactic properties of Si against powdery mildews on a number of crops (Chérif et al., 1992; Dik et al., 1998; Bélanger et al., 2003; Shetty et al., 2012). In a recent study, Vivancos et al. (2015) suggested that Si was particularly efficient against biotrophic pathogens because of the mode of attack of these pathogens relying on effectors to establish biotrophy; Si deposition would interfere with effectors finding their specific targets. Regardless of the mode of action, our data show convincingly that Si can be an efficient treatment to reduce powdery mildew severity in strawberry. Given that powdery mildew becomes particularly severe in late summer and early fall under our tested conditions, and that strawberry plants continue to absorb Si actively from July to September, there is a positive synchrony between the treatment and disease reduction. Unfortunately, both the absence of powdery mildew infection and the lack of an efficient Si treatment in 2015 and 2016, prevent from drawing conclusions about Si efficacy in the field experiments.

The reduction in powdery mildew severity linked to Si fertilization in high tunnels translated into significantly higher yields in terms of marketable fruits in both seasons and all cultivars. While it is unknown if the yield increase is solely attributable to powdery mildew reduction, or to other stimulating factors linked to Si, these positive effects were quite remarkable in some cultivars (e.g., Monterey) and certainly represent the highest selling point for growers to implement this approach. In addition, our data never showed negative effects of Si on plants or fruits, thus confirming the lack of phytotoxicity of the element. These observations allow to reject claims linking soluble Si fertilization to albino fruits (Lieten et al., 2002), an artifact most likely attributable to improper mixing of Si in the nutrient solution. For a producer, the main objective remains to optimize yield in order to maximize profits. If this can be attained by reducing the negative environmental impact of fungicides used for powdery mildew control, Si fertilization could find a niche as a component of an integrated program for strawberry production.

## Conclusions

In conclusion, the objectives of this project were to determine if strawberry had the proper genetic tools to absorb Si, and to determine the fertilization conditions under which strawberry could benefit from a Si treatment. Our results showed that strawberry has the proper transporters to uptake Si, and that under a constant soluble Si fertilization regime and a peat substrate, it can absorb as much as 3% Si d.w., clearly classifying it as a Si-competent species. This treatment reduced significantly the severity of *P. aphanis* in all tested cultivars and increased the yield of marketable fruits. These results highlight the potential of Si amendments for producers with opportunities to lower fungicide use and increase revenues. Our results further show that the fertilization regimes and soil conditions can greatly influence the benefits strawberry will obtain from Si amendments, and these should be considered carefully before fertilizing with Si.

## Author contributions

SO and MG performed all the greenhouse and field experiments, CL participated in greenhouse experiment and silicon quantification, CL and JL involved in data analysis. RD performed the bioinformatic analysis, LG, AG, MD involved in planning the field and greenhouse experiments, RB designed and directed the project. All authors contributed in drafting the MS.

### Conflict of interest statement

The authors declare that the research was conducted in the absence of any commercial or financial relationships that could be construed as a potential conflict of interest.
