# Supplementary material for: Silicon Transporters and Effects of Silicon Amendments in Strawberry under High Tunnel and Field Conditions
Source: Front Plant Sci. 2017 Jun 8;8:949. doi: 10.3389/fpls.2017.00949 (PMC5462948; doi:10.3389/fpls.2017.00949)
Supplement: Supplementary file 1 [file Table1.DOCX]

**Supplementary Table 1** Functional classification of FaLsi2 identified in strawberry based on subfamily domain architectures performed using CDD search tool hosted at NCBI*

|  | **Name** | **Accession** | **Description** | **Interval** | **E-value** |
| --- | --- | --- | --- | --- | --- |
| **1** | PLN00136 | [PLN00136](https://www.ncbi.nlm.nih.gov/Structure/cdd/cddsrv.cgi?ascbin=8&maxaln=10&seltype=2&uid=PLN00136) | silicon transporter; Provisional | 1-542 | 0e+00 |
| 2 | YbiR_permease | [cd01117](https://www.ncbi.nlm.nih.gov/Structure/cdd/cddsrv.cgi?ascbin=8&maxaln=10&seltype=2&uid=cd01117) | Putative anion permease YbiR. Based on sequence similarity, YbiR proteins | 28-542 | 7.06e-113 |
| 3 | CitMHS | [pfam03600](https://www.ncbi.nlm.nih.gov/Structure/cdd/cddsrv.cgi?ascbin=8&maxaln=10&seltype=2&uid=pfam03600) | Citrate transporter; | 32-231 | 7.61e-45 |
| 4 | ArsB | [COG1055](https://www.ncbi.nlm.nih.gov/Structure/cdd/cddsrv.cgi?ascbin=8&maxaln=10&seltype=2&uid=COG1055) | Na+/H+ antiporter NhaD or related arsenite permease [Inorganic ion transport and metabolism]; | 15-542 | 1.10e-46 |
| 5 | PLN00137 | [PLN00137](https://www.ncbi.nlm.nih.gov/Structure/cdd/cddsrv.cgi?ascbin=8&maxaln=10&seltype=2&uid=PLN00137) | NHAD transporter family protein; Provisional | 391-500 | 8.35e-06 |

*https://www.ncbi.nlm.nih.gov/Structure/cdd/cdd.shtml
