# Supplementary figures and images for: Silicon Transporters and Effects of Silicon Amendments in Strawberry under High Tunnel and Field Conditions
Source: Front Plant Sci. 2017 Jun 8;8:949. doi: 10.3389/fpls.2017.00949 (PMC5462948; doi:10.3389/fpls.2017.00949)

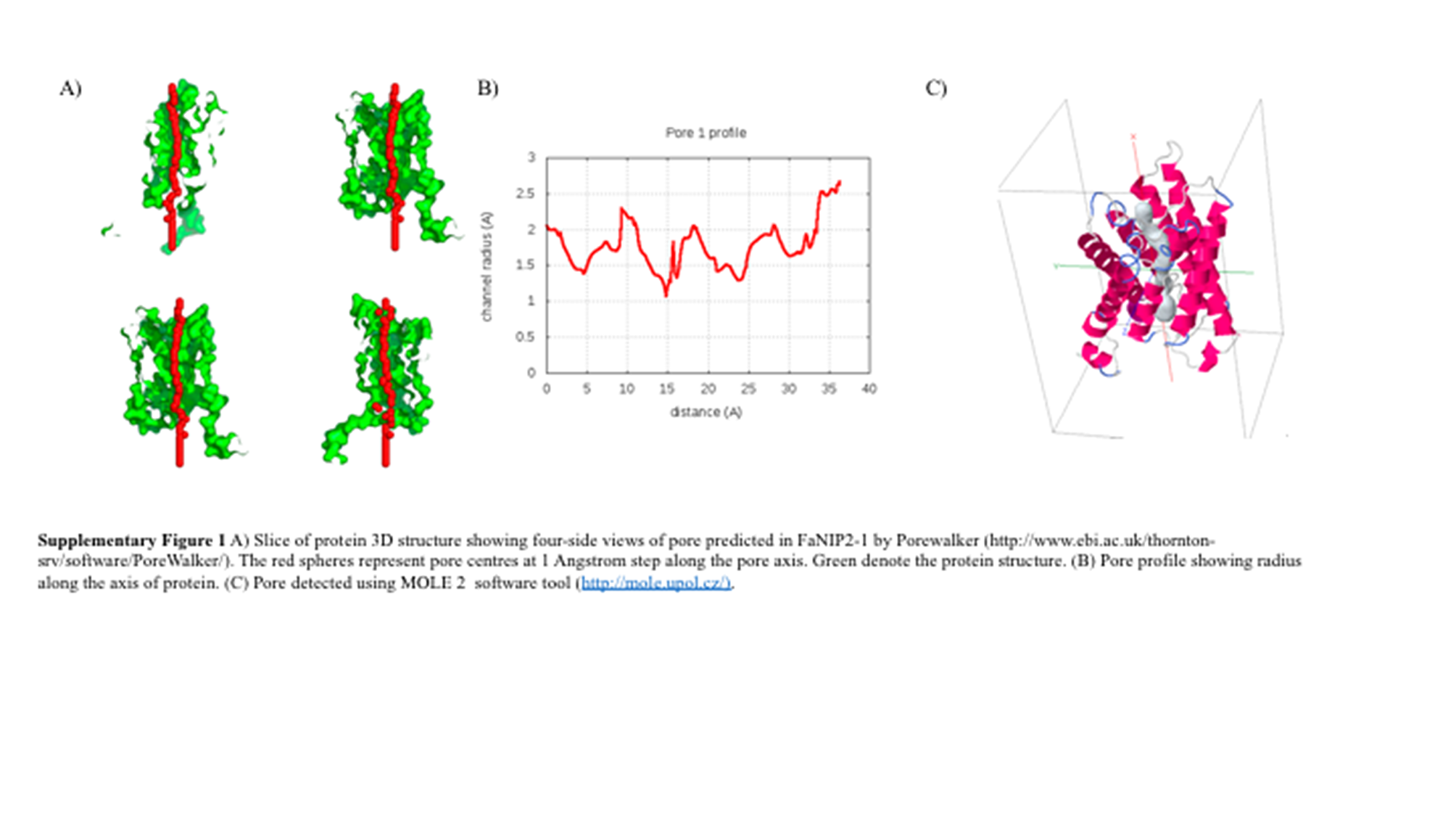

Supplement: Supplementary file 3 [file Image1.TIFF]
